# Rationale and protocol for estimating the economic value of a multicomponent quality improvement strategy for diabetes care in South Asia

**DOI:** 10.1186/s41256-019-0099-x

**Published:** 2019-03-18

**Authors:** Kavita Singh, Mohammed K. Ali, Raji Devarajan, Roopa Shivashankar, Dimple Kondal, Vamadevan S. Ajay, V. Usha Menon, Premlata K. Varthakavi, Vijay Viswanathan, Mala Dharmalingam, Ganapati Bantwal, Rakesh Kumar Sahay, Muhammad Qamar Masood, Rajesh Khadgawat, Ankush Desai, Dorairaj Prabhakaran, K. M. Venkat Narayan, Victoria L. Phillips, Nikhil Tandon, On behalf of the CARRS Trial Group

**Affiliations:** 1 Public Health Foundation of India, Center of Excellence - Center for CArdio-metabolic Risk Reduction in South Asia, 4th Floor, Plot No. 47, Sector 44, Institutional Area, Gurgaon, Haryana 122 002 India; 2 Rollins School of Public Health, Emory University, 1518 Clifton Road, Rm CNR 7041, Atlanta, GA 30322 USA; 3 Department of Endocrinology & Diabetes, Amrita Institute of Medical Sciences, Amrita Vishwa Vidyapeetham, Ponekkara P.O., Kochi, Kerala 682 041 India; 4 Department of Endocrinology, TNM College & BYL Nair Charity Hospital, Dr. A. L. Nair Road, Mumbai Central, Mumbai, Maharashtra 400 008 India; 5 MV Hospital for Diabetes & Diabetes Research Centre, No 4, West Madha Church Street, Royapuram, Chennai, Tamil Nadu 600 013 India; 6 Bangalore Endocrinology & Diabetes Research Centre, #35, 5th Cross, Malleswaram Circle, Bangalore, Karnataka 560 003 India; 7 Department of Endocrinology, St. John’s Medical College & Hospital, Sarjapur Road, Koramangala, Bangalore, Karnataka 560 034 India; 8 Department of Endocrinology, Osmania General Hospital, 2nd Floor, Golden Jubilee Block, Afzalgunj, Hyderabad, Telangana 500 012 India; 9 Department of Medicine, Section of Endocrinology and Diabetes, Aga Khan University, Stadium Road, Karachi, 74800 Pakistan; 10 Department of Endocrinology & Metabolism, All India Institute of Medical Sciences, Biotechnology Block, 3rd Floor, Ansari Nagar, New Delhi, 110 029 India; 11 Endocrine Unit, Department of Medicine, Goa Medical College, Bambolim, Goa 403202 India; 12 Public Health Foundation of India, 4th Floor, Plot No. 47, Sector 44, Institutional Area, Gurgaon, Haryana 122 002 India; 13 Rollins School of Public Health, Emory University, 1518 Clifton Road, Rm CNR 7049, Atlanta, GA 30322 USA; 14 Rollins School of Public Health, Emory University, 1518 Clifton Road, Atlanta, GA 30322 USA; 15 Department of Endocrinology & Metabolism, All India Institute of Medical Sciences, Biotechnology Block, 3rd Floor, Rm #312, Ansari Nagar, New Delhi, 110 029 India; 16 Centre for Chronic Disease Control, C 1/52, Second Floor, Safdarjung Development Area, New Delhi, 110016 India; 17 London School of Hygiene and Tropical Medicine, Keppel St, Bloomsbury, London, WC1E 7HT UK

**Keywords:** Economic evaluation, Diabetes care, Multicomponent strategy, Quality improvement, South Asia

## Abstract

**Background:**

Economic dimensions of implementing quality improvement for diabetes care are understudied worldwide. We describe the economic evaluation protocol within a randomised controlled trial that tested a multi-component quality improvement (QI) strategy for individuals with poorly-controlled type 2 diabetes in South Asia.

**Methods/design:**

This economic evaluation of the Centre for Cardiometabolic Risk Reduction in South Asia (CARRS) randomised trial involved 1146 people with poorly-controlled type 2 diabetes receiving care at 10 diverse diabetes clinics across India and Pakistan. The economic evaluation comprises both a within-trial cost-effectiveness analysis (mean 2.5 years follow up) and a microsimulation model-based cost-utility analysis (life-time horizon). Effectiveness measures include multiple risk factor control (achieving HbA1c < 7% and blood pressure < 130/80 mmHg and/or LDL-cholesterol< 100 mg/dl), and patient reported outcomes including quality adjusted life years (QALYs) measured by EQ-5D-3 L, hospitalizations, and diabetes related complications at the trial end. Cost measures include direct medical and non-medical costs relevant to outpatient care (consultation fee, medicines, laboratory tests, supplies, food, and escort/accompanying person costs, transport) and inpatient care (hospitalization, transport, and accompanying person costs) of the intervention compared to usual diabetes care. Patient, healthcare system, and societal perspectives will be applied for costing. Both cost and health effects will be discounted at 3% per year for within trial cost-effectiveness analysis over 2.5 years and decision modelling analysis over a lifetime horizon. Outcomes will be reported as the incremental cost-effectiveness ratios (ICER) to achieve multiple risk factor control, avoid diabetes-related complications, or QALYs gained against varying levels of willingness to pay threshold values. Sensitivity analyses will be performed to assess uncertainties around ICER estimates by varying costs (95% CIs) across public vs. private settings and using conservative estimates of effect size (95% CIs) for multiple risk factor control. Costs will be reported in US$ 2018.

**Discussion:**

We hypothesize that the additional upfront costs of delivering the intervention will be counterbalanced by improvements in clinical outcomes and patient-reported outcomes, thereby rendering this multi-component QI intervention cost-effective in resource constrained South Asian settings.

**Trial registration:**

ClinicalTrials.gov: NCT01212328.

## Background

Diabetes is one of the fastest growing public health problems with huge financial burdens. The global costs of diabetes were US$ 1.31 trillion (1.8% of global GDP) in 2015 [1]. A 2018 systematic review found that annual costs of diabetes care (out of pocket medical expenditure) in South Asia ranged between US$ 575 to US $1216 per person [2]. Diabetes is a progressive disease which requires increasingly more clinic visits, laboratory tests, and patients need to engage with the healthcare system and providers over years for better management of diabetes which can arrest disease progression. However, current chronic care for diabetes is sub-optimal, costly, and lower socioeconomic status or uninsured individuals may be more likely to experience poor control [3–7].

Several barriers at the patient- (e.g., low motivation, financial barriers), provider- (e.g., inertia to intensify treatments), and system-level (e.g., complicated and/or fragmented care system), individually or together, cause patient and system “fatigue” and disrupt achievement of diabetes care goals [8–10]. In the Centre for Cardio-metabolic Risk Reduction in South Asia (CARRS) Trial, we targeted different levels of barriers together (e.g., patient motivation and provider inertia) [9, 11] and demonstrated sustainable and larger improvements in outcomes and satisfaction for people with diabetes with a multicomponent strategy of decision support-electronic health records (DS-EHR) and non-physician care coordinators (CC), compared to usual diabetes care [12].

However, enhancements or changes to the status quo of care delivery come at a cost, and in order to formulate useful recommendations for practicing clinicians, health systems, payers (health insurance, governments, patients paying out-of-pocket), and policymakers, there is an imperative to assess the value of investing in quality improvement (QI) care models. Knowing the upfront costs is also necessary to guide decision makers as they consider implementation of QI interventions in clinical care.

A 2018 systematic review of economic evaluations of QI interventions for glycaemic control among adults with type 1 or type 2 diabetes from high income countries found that multifaceted QI interventions that lower HbA1c was good value for money versus usual care, depending on society’s willingness to pay [13]. However, in our review of cost-effectiveness of interventions to control cardiovascular diseases and diabetes mellitus, we found a scarcity of cost-effectiveness studies related to QI interventions for diabetes care in South Asia [14]. Here, we describe the economic evaluation protocol to assess the within-trial cost-effectiveness and broader societal value of the CARRS diabetes care model consisting of DS-EHR and non-physician CCs compared to usual diabetes care.

## Methods/Design of Economic Evaluation

### Overview

The objectives of the economic evaluation are to assess: a) the incremental cost of delivering multicomponent QI interventions compared to usual diabetes care in tertiary care settings over a period of 2.5 years; b) whether the intervention provides value for money (cost-effectiveness) to patients, healthcare systems and society than usual care, and if so; c) the extent of uncertainty over the cost-effectiveness of the intervention and value of conducting further research to reduce this uncertainty.

The CARRS Trial’s economic evaluation will follow standard international methodological guidelines [15–18]. Given, more than 80% of medical expenses in India and Pakistan are out-of-pocket expenditures borne by the patient, we will apply the patient viewpoint as the predominant perspective, in addition to healthcare system and societal perspectives for costing resource use. Cost data will be reported in 2018 United States Dollars (US$). Both cost and health effects will be discounted at 3% per year as per the World Health Organization’s (WHO) guidelines for conducting economic evaluations in developing countries.

### The CARRS trial and study population

The CARRS Trial randomised 1146 eligible patients with poorly controlled type 2 diabetes (HbA1c > 8% and SBP > 140 mmHg or LDLc> 130 mg/dl) to intervention (*n* = 575) or usual care (*n* = 571) across 10 diverse diabetes clinics in India and Pakistan. At baseline, participants’ mean age was 54 years, 45% were males, mean HbA1c was 9.9%, LDLc 123.2 mg/dl, BP 144.2/82.3 mmHg, and median duration of diabetes was 7 years [12].

### Intervention and comparator

Detailed information about the CARRS-Trial intervention and protocol has been published previously [19]. Briefly, the CARRS intervention consisted of DS-EHRs to enhance physicians’ responsiveness to consider treatment modification and non-physician CCs to support patients in their adherence to prescribed therapies. The DS-EHR stored all consultation, laboratory, self-care, and diabetes related complications data for patients in one easily accessible web portal to monitor patient progress; and provided decision-support system (DSS) prompts to facilitate achievement of guideline-recommended glycemic, blood pressure, and lipid goals. The CCs fully managed the DS-EHR data-entry for intervention group participants and all communication of DSS prompts to the physician during consultations via print-out or electronic display. Physicians could, at their discretion, accept or reject DSS prompts and modify treatment plans based on clinical judgment, so long as justification was provided.

**Fig. 1:**
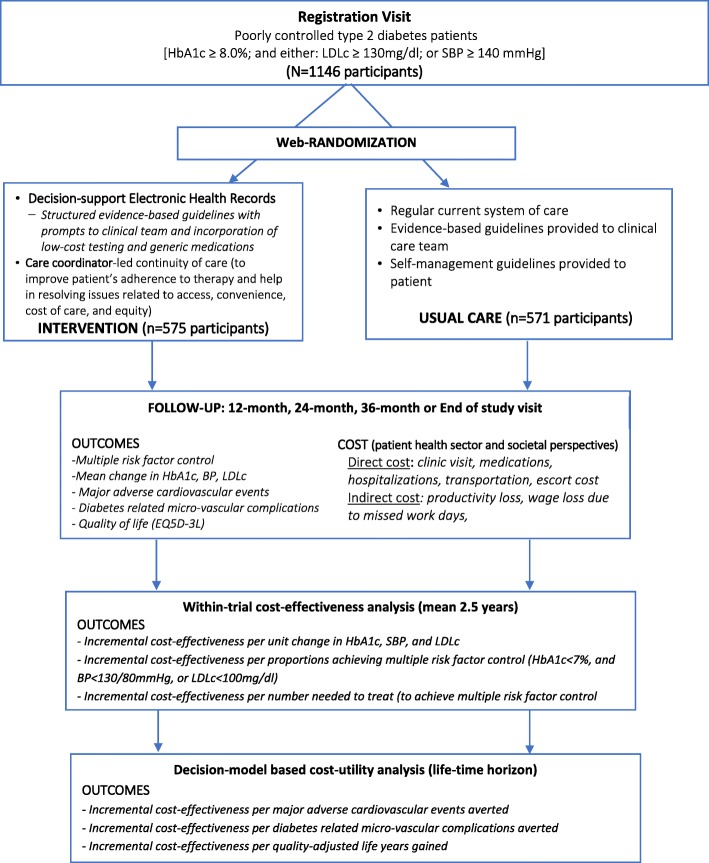
Study flow - Economic evaluation alongside CARRS Trial. Abbreviations: CARRS=Centre for Cardiometabolic Risk Reduction in South Asia), DS-EHR = decision-support electronic health records; HbA1c = glycated hemoglobin, BP = blood pressure, SBP = systolic blood pressure, LDLc = low-density lipoprotein cholesterol, EQ5D-3 L = European quality of life 5 dimension 3 levels; mg/dl = milligrams per deciliter, mmHg = millimeter of mercury

The intervention was compared with usual diabetes care at nine clinics/hospitals across India and one site in Pakistan. Figure 1 demonstrates the study flow.

### Effectiveness measures

To evaluate incremental effectiveness, we will compare the proportions of intervention and control arm participants achieving multiple risk factor control defined as HbA1c < 7% and BP < 130/80 mmHg or LDLc< 100 mg/dl (and < 70 mg/dl for those with history of cardiovascular disease). Data on health-related quality of life (EQ5D-3 L); new-onset cardiovascular events, new onset microvascular events, and other hospitalizations would also be used.

The CARRS Trial is currently ongoing and we will project cardiovascular and microvascular outcomes using proxy indicators (intermediate risk factors: HbA1c, BP, LDLc). Relative risk reductions for major adverse cardiovascular events with intervention or comparator will also be calculated separately for each participant, using United Kingdom Prospective Diabetes Study (UKPDS) Outcomes Models 2 which has been validated for use in South Asians [20]. Table 1 summarizes the study outcomes (effectiveness measures) to be considered in the cost-effectiveness analysis.

**Table 1:** Overview of the effectiveness measures

Measure	Means of collection	Timing of collection	Source of data
Multiple risk factor control (HbA1c < 7% and BP < 130/80 mmHg or LDLc< 100 mg/dl)	Blood test + BP measurement using digital BP monitor	Baseline: Prior to intervention deliveryFollow-up: Annual visits post intervention delivery	Trial eCRF (Form C, E, F)
Single risk factor control			
HbA1c (1% point reduction)	Fasting blood test	Baseline: Prior to intervention deliveryFollow-up: Annual visits post intervention delivery	Trial eCRF (Form C, E, F)
SBP (5 mmHg reduction)	BP measurement using digital BP monitor (Omron-T9P)	Baseline: Prior to intervention deliveryFollow-up: Annual visits post intervention delivery	Trial eCRF (Form C, E, F)
DBP (5 mmHg reduction)	BP measurement using digital BP monitor (Omron-T9P)	Baseline: Prior to intervention deliveryFollow-up: Annual visits post intervention delivery	Trial eCRF (Form C, E, F)
LDLc (10 mg/dl reduction)	Fasting blood test	Baseline: Prior to intervention deliveryFollow-up: Annual visits post intervention delivery	Trial eCRF (Form C, E, F)
Major adverse cardiovascular events	Self-reported by patient and physician verified	Follow-up: All study related and non-study related clinic visits	Trial eCRF (form X)
Diabetes related micro-vascular complications	Self-reported by patient and physician verified	Follow-up: All study related and non-study related clinic visits	Trial eCRF (form X)
Quality adjusted life years	EQ5D-3 L	Baseline: Prior to intervention deliveryFollow-up: Annual visits post intervention delivery	Trial eCRF (Form C, E, F)

*HbA1c* Glycated haemoglobin, *SBP* Systolic blood pressure, *LDLc* Low-density lipoprotein cholesterol, *eCRF* Electronic case report form, *EQ5D-3 L* European Quality of Life five dimension 3 levels, *BP* Blood pressure, *mg/dl* Milligrams per deciliter, *mmHg* Millimeter of mercury

### Resource use and cost data

Resource utilization and costs will be estimated using data from the CARRS Trial population (1146 participants). The study paid the costs of annual laboratory investigations, but patients had to bear the cost of clinic visits and laboratory tests for regular follow-up visits or any other interim clinic visits, tests, medication changes, or procedures advised by the treating physician. CARRS Trial data will be extracted from clinic and study records for the following: medication use, laboratory tests, consultations with healthcare professionals (outpatient attendance for diabetes); preventive screening (eye examination, foot examination, ECG, microalbuminuria test), emergency department attendances (when not admitted to hospital); and serious adverse events (including all hospital admissions).

Patients’ self-reported expenditures and costs of outpatient visits and hospitalizations related to diabetes complications will be extracted from the trial annual visit case report forms (CRF). Out-of-pocket expenses reported by the patients will permit estimation of economic value from the patient’s perspective.

To estimate value from a healthcare system perspective, unit costs for outpatient visits and in-patient hospitalizations, and processes of care measures including preventive examinations will be obtained from participating hospitals. For treatment of cardiovascular and microvascular events, we will extract detailed information concerning diagnosis; length of hospital stay; diagnostic/therapeutic procedures and any ongoing treatment and support. Additionally, the unit price of medications will be obtained from the PharmaTrac database for January 2014 [21]. PharmaTrac provides the market retail price (MRP) of all drugs by drug class, brand name, generic composition, formulation (oral/injectables), dose, and packs being sold in India. PharmaTrac has an extensive coverage of drug retailers and is believed to be a reliable source to estimate unit cost of drug prices in India. The IMS Health drug database will be used to estimate drug prices in Pakistan.

To estimate costs from the societal perspective, indirect costs due to lost productivity (number of work days missed due to out-patient or in-patient care) will be valued using the human capital approach [22]. Finally, total costs over the trial period and annual cost per patient (both undiscounted and discounted) will be estimated for individual patients by multiplying resource use by unit costs.

### Intervention costs

Intervention development and delivery costs will be derived from the CARRS Trial expense records (accounts register) and will be estimated from the health system perspective. Intervention costs include DS-EHR development, implementation, and maintenance costs; intervention training; care coordinator salary; and the incremental health care costs associated with the intervention delivery (i.e. the costs of additional medicines, additional clinic visits that patients bear and whether it is different between the treatment groups). These costs will be calculated as average costs of implementation per person and exclude any research specific costs. The cost estimates assume that the DS-EHR is implemented in a relatively large tertiary care hospital having additional resource facilities to implement the intervention (i.e. workspace for the care coordinator, and access to internet service providers is considered a maintenance cost). DS-EHR development and maintenance costs will include software programmer’s time, expert consultant’s/physicians time in developing and reviewing the diabetes management algorithm. DS-EHR implementation cost will include care coordinators and site physician’s time to enter patient details in the EHR system and review of software generated diabetes management plans, respectively. Intervention training costs include training materials, the time of the trainers and the staff participating in the training, and training for physicians to use the DS-EHR algorithms. These costs will be estimated using the study’s accounting data. Training material and time costs will be estimated from the first year of the intervention. Tables 2 and 3 present an overview of cost measures, health service use, and source of data.

**Table 2:** Overview of cost measures

Type of cost	Level	Expense type	Cost component	Means of collection	Timing of collection	Source of data
Direct	Intervention	Fixed cost	Software development	Trial records	After completion of software development	Developers of software: DS-EHR
		Fixed cost	Training of physicians and care coordinators	Trial records	After completion of training	Trial Team
		Fixed cost	Laptop	Trial records	Baseline (at the start of the trial)	Trial records
		Fixed cost	Mobile phone	Trial records	Baseline (at the start of the trial)	Trial records
		Variable cost	Care coordinator’s salary	Trial records	Monthly	Payment invoice
		Variable cost	Three-monthly laboratory tests	Interview with patients	Annual	Self-reported by patient
		Variable cost	Internet	Trial records	Annual	Payment invoice
		Variable cost	Communication cost	Trial records	Annual	Payment invoice
		Fixed cost	Software maintenance	Trial records	Annual	Payment invoice
	Clinic/Hospital	Variable cost	Physician’s time	Interview with patients and physicians	Annual	Self-reported by physicians
		Variable cost	Resource use for patient management: telephone calls, letters, team meetings	Interview with physicians and hospital administrators	Annual	Self-reported by administrators
	Patient	Variable cost	Medications	Interview with patients + Trial eCRF	Annual	Self-reported by patient in eCRF
		Variable cost	Medical supplies (glucose strips, gauze, sterile solution, etc.)	Interview with patients + Trial eCRF	Annual	Self-reported by patient in eCRF
		Variable cost	Laboratory tests	Interview with patients + Trial eCRF	Annual	Self-reported by patient in eCRF
		Variable cost	Diagnostics	Interview with patients + Trial eCRF	Annual	Self-reported by patient in eCRF
		Variable cost	Preventative screening (ECG, eye exam, foot exam, dental exam, etc.)	Interview with patients + Trial eCRF	Annual	Self-reported by patient in eCRF
		Variable cost	Outpatient visits (consultation fee)	Interview with patients + Trial eCRF	Annual	Self-reported by patient in eCRF
		Variable cost	Transportation	Interview with patients + Trial eCRF	Annual	Self-reported by patient in eCRF
		Variable cost	Food (personal)	Interview with patients + Trial eCRF	Annual	Self-reported by patient in eCRF
		Variable cost	Additional cost for escort	Interview with patients + Trial eCRF	Annual	Self-reported by patient in eCRF
		Variable cost	Other out of pocket expenses	Interview with patients + Trial eCRF	Annual	Self-reported by patient in eCRF
		Variable cost	In-patient hospitalization	Interview with patients + Trial eCRF	Annual	Self-reported by patient in eCRF
		Variable cost	Procedures	Interview with patients + Trial eCRF	Annual	Self-reported by patient in eCRF
Indirect	Patient	Variable cost	Lost-productivity	Interview with patients + Trial eCRF	Annual	Self-reported by patient in eCRF
		Variable cost	Work days lost due to out-patient visit	Interview with patients + Trial eCRF	Annual	Self-reported by patient in eCRF
		Variable cost	Work days lost due to in-patient hospitalization	Interview with patients + Trial eCRF	Annual	Self-reported by patient in eCRF
		Variable cost	loss of concentration	Interview with patients + Trial eCRF	Annual	Self-reported by patient in eCRF
		Variable cost	loss of function (health utility index)	Interview with patients + Trial eCRF	Annual	Self-reported by patient in eCRF

*DS-EHR* Decision-support Electronic Health Records, *ECG* Electrocardiogram, *eCRF* Electronic case report form

**Table 3:** Self-reported health services use and sources of data

Service type	Source of unit costs
Outpatient clinic visit	OOP
HbA1c testing	OOP
Cholesterol testing	OOP
Foot examination at clinic	OOP
Eye examination	OOP
Microalbuminuria check	OOP
ECG	OOP
Dental exam	OOP
Dietician visit	OOP
Diabetes educator visit	OOP
Other healthcare practitioner visits	OOP
Time spent commuting to clinic	Wage loss due to missed work days
Time spent for lab tests including waiting	Wage loss due to missed work days
Time spent waiting for consultation	Wage loss due to missed work days
Time spent in-person with doctor	Wage loss due to missed work days
Time spent with dietician, nurse of clinic-staff receiving self-care education	Wage loss due to missed work days
Other time for check-out including medications	Wage loss due to missed work days
Hospitalization	OOP
Emergency room visit	OOP
Medications	Pharmatrac/IMS/OOP

*Abbreviations*: *OOP* Out of pocket medical expenses, *HbA1c* Glycated haemoglobin, *ECG* Electrocardiogram

### Within trial cost-effectiveness analysis

Based on estimates of between-group differences in mean healthcare costs and outcomes (adjusting for differences in baseline characteristics) over the study period, we will estimate the following incremental cost-effectiveness ratios (ICERs):

- Incremental cost per primary outcome achieved (i.e. multiple risk factor control: HbA1c < 7% and BP < 130/80 mmHg and/or LDLc< 100 mg/dl)
- Incremental cost per unit reduction in single risk factors: HbA1c (1% point reduction), SBP (5 mmHg reduction), and LDLc (10 mg/dl reduction)
- Incremental cost per quality adjusted life years (QALYs) gained

Non-parametric bootstrapping will be used to report 95% confidence intervals around the ICER estimates [23]. ICERs will be reported in US$ 2018. Cost effectiveness acceptability curves against a wide range of willingness to pay values will be presented [24]. Cost-effectiveness results will be also presented by major sub-groups: age, gender, education, income level, types of health setting (public, private or semi-private) and history of macro- and micro-vascular complications.

### Missing data

The CARRS Trial has a minimal loss to follow-up including consent withdrawals and deaths at 2.5 years (9.2%) but, if needed; multiple imputation approaches will be used to handle missing outcomes data [12]. For EQ5D-3 L scores, which will be used for QALY estimation, we will follow the developer’s guideline for missing data; that is, missing data will be handled by imputing values within each dimension [25–28]. To address potential biases due to incomplete follow-up, we will use multiple imputation approaches to replace missing cost values if missing data accounts for more than 10% of a domain/variable [29–31]. Since cost data are unlikely to be normally distributed, [29] we will use the multiple imputation chained equations approach to impute missing cost data. Costs will be imputed at the total cost level [29].

### Decision-modeling based cost-utility analysis

A decision-analytic microsimulation model will be developed to evaluate long-term costs and health consequences of delivering care for people with type 2 diabetes using a multicomponent QI strategy rather than current standard care approaches. A microsimulation model is chosen as it is very flexible and can reflect complex treatment pathways and relationships between individuals’ characteristics, histories, and outcomes; it can be used to examine the impact of real resource constraints within a healthcare system.

The microsimulation decision model will be implemented using appropriate software: STATA or a programming language (e.g. R). To assure the credibility of our model, we will follow international guidelines for verification and validation of decision models [32].

### Model analysis

All analyses will compare results for the CARRS multicomponent QI care delivery model versus usual diabetes care. In the CARRS Trial microsimulation model, costs and QALYs will be recorded for each individual and an average cost and QALY for the simulated population will be estimated. The microsimulation model will be run twice, once to simulate costs and QALYs under usual care and the other to simulate costs and QALYs under the intervention scenario (multicomponent QI strategy). Individuals representing the CARRS trial inclusion criteria will enter the model and their baseline risk for CVD events and diabetes-related microvascular complications will be estimated using the UKPDS Outcomes Model 2 algorithm. Costs and QALYs will be recorded for each event (including adverse events). Individuals can experience more than one event (model run for lifetime horizon) and patient characteristics such as age and history of previous events, such as a stroke or diabetic retinopathy, will be updated as the model is being run, with ensuing reflective increases in the risk of an event. The simulation model will run for a sufficient number of iterations to provide stable results. If there is a trade-off between costs and health effects (higher costs and better health outcomes for the CARRS intervention, or vice versa), the incremental cost per cardiovascular event averted, incremental cost per diabetes-related microvascular complication averted, and incremental cost per quality adjusted life year (QALY) gained will be reported. Projections of cost-effectiveness estimates over a lifetime horizon will be made for India and Pakistan.

### Sensitivity analysis

Several one-way sensitivity analyses will be carried out to estimate the uncertainties around ICERs. First, to address the uncertainty around the ICER relating to external validity, we will carry out sensitivity analyses on the most important cost drivers (medications, hospitalizations, and consultation fees) to assess the impact of protocol-driven healthcare use. Second, total cost will be calculated with and without the costs of developing the intervention (DS-EHR) to ascertain whether an increased cost in the intervention arm could be explained by costs for some of the components of the intervention. Lastly, sensitivity analyses would vary the effectiveness of the intervention in trial vs. non-trial settings based on the lower and upper limit of 95% confidence intervals (CI) of the effect estimates. Results of probabilistic sensitivity analyses will be presented using a scatter plot of points on the cost-effectiveness plane – illustrating the possible ranges of estimates of incremental costs and incremental QALYs [24].

## Discussion

The publication and peer-review of economic evaluation protocols alongside clinical trials is recommended to increase transparency and minimise bias [33]. Here, we describe the protocol of an economic evaluation of a multicomponent QI strategy compared to usual diabetes care in South Asia from patient, healthcare system, and societal perspectives. There are very few economic evaluations of QI strategies for chronic disease management in South Asia [34] or in LMICs in general, and so this report fills a gap. Following internationally recognised guidelines [15], this protocol serves to heighten the transparency of our economic evaluation approach.

Economic evaluations from high-income countries demonstrate that multifactorial QI strategies are cost-effective. For example, the STENO-2 study showed that, from a health care payer perspective in Denmark, intensive multifactorial intervention was more cost-effective than conventional treatment (ICER: €2538 or US$ 2954 per QALY gained) over a lifetime horizon [35]. Increased costs with intensive treatment were due to increased pharmacy and consultation costs. However, this also resulted in more QALYs gained for intensive treatment versus conventional treatment (+ 1.66 QALYs). The ADDITION-UK trial based cost-effectiveness analysis comparing intensive versus conventional treatment demonstrated an ICER of £71,232 (US$93566)/QALY, £28,444 (US$37362)/QALY, and £27,549 (US$36186)/QALY over 10-, 20-, and 30-year time horizons respectively [36]. Given the United Kingdom’s willingness-to-pay thresholds in patients with diabetes, intensive treatment was of borderline cost-effectiveness over a time horizon of ≥20 years. The estimates of cost-effectiveness from the CARRS Trial will provide much needed data on whether a simple multifactorial intervention can improve health outcomes with modest increases in costs in resource-constrained settings.

To enhance external validity, it is recommended that evaluations using randomised controlled trials should identify threats to validity such as recruitment/selection bias, protocol-driven utilisation, and enhanced compliance [34, 37]. Regarding recruitment biases, the CARRS Trial’s multicentre approach and inclusion of public, private, and semi-private practices increases the generalisability and transferability of our economic evaluation findings [38]. Further, we will extrapolate the decision analytic microsimulation model beyond the within-trial analysis by using a sample population of poorly controlled type 2 diabetes patients in India / Pakistan stratified by age-group, gender, and location. Also, although Markov models can also be adapted for this purpose, microsimulation models are better suited for analysis of a mixed population with both incident and prevalent diabetes complications (cardiovascular diseases and microvascular events) [39].

This study has several strengths. First, the economic evaluation protocol follows recognised international guidelines to design and report on the relative costs and benefits of an intervention tested in a randomised trial [15, 37]. Second, the economic evaluation will include individual patient-level data over a lengthy 2.5 years of follow up, which are preferable for economic evaluations [15]. Importantly, these patient-level data include objective measures of health outcomes, health service use, and medicine use, all obtained during the trial [40]. Reliable economic evaluations are crucial to shape healthcare policy, in particular when the possibility of bias in economic evidence has been minimised by randomisation [40]. Third, our cost-effectiveness results will also provide a range of values for both the cost of achieving multiple risk factor targets but also costs to achieve single and combined risk factor improvements from poorly controlled baseline values (mean baseline HbA1c = 9.9) from various perspectives (patient, healthcare system and societal). Given a large proportion of healthcare in South Asia is paid for out-of-pocket, our economic analyses consider that scenario explicitly with a patient perspective analysis. Fourth, our proposed micro-simulation model based on UKPDS Outcomes Model 2 will enable long-term cost-effectiveness analysis and a population budget impact analysis which will provide cross-sectional estimates of population impact by year for planning purposes and scalability of the intervention.

This study has a few noteworthy limitations. First, reliance on patient self-reported out-of-pocket medical cost data may impact the validity of study results. A 2016 systematic review of validated self-reported questionnaires to measure resource utilization and costs in economic evaluation concluded that self-reported questionnaires had good agreement with administrative data and are a valid method of collecting data on health resource utilization and associated costs [41]. However, to overcome any reporting bias in self-reported costs data, a sub-set of self-reported costs will be verified against the administrative data and we will carry out several one-way and probabilistic sensitivity analyses around the self-reported costs in the microsimulation model to estimate the confidence in the reported ICER values. Another limitation of the proposed evaluation is that in India and Pakistan, there is not an explicit willingness to pay threshold for reduction of cardiovascular risk in people with diabetes, or an explicit willingness to pay threshold for cost per unit reductions in CVD risk factors. As such, it is hard to declare how patients value the intervention. The Commission for Macroeconomics and Health recommends using a threshold of 1-3x GDP per capita per QALY gained or DALYs averted to define cost-effectiveness of a new intervention when conducting global or regional economic evaluations [16, 42]. Although arbitrary, we will use this threshold as it has been used previously and has some philosophical underpinnings [43]. We will apply these and then perform a sensitivity analysis for the main economic outcome and present the cost-effectiveness results on a cost-effectiveness acceptability curve considering a wide range of willingness to pay values. Common to all cost-effectiveness analyses conducted alongside randomised trials, external validity of the results may be influenced by restrictive inclusion criteria and protocol-driven resource use, among other factors [15, 37]. Therefore, we will conduct a range of sensitivity analyses around key variables (cost drivers, total cost calculated with and without the cost of the intervention development, patient characteristics, and effectiveness of the intervention) to address the uncertainties around the ICERs.

In conclusion, we hypothesise that the additional upfront cost of delivering the intervention will be counterbalanced by improvements in clinical practice and patient related outcomes, thereby rendering the CARRS QI strategy cost-effective. The results of this study will be of immediate relevance for decision makers of all sorts –patients, healthcare providers, and policy makers– concerning implementation of this healthcare delivery intervention to improve diabetes care goals.

## Abbreviations

€: Euros
BP: Blood pressure
CARRS: Centre for Cardiometabolic Risk Reduction in South Asia
CC: Care coordinator
CI: Confidence interval
CRF: Case report form
CVD: Cardiovascular disease
DBP: Diastolic blood pressure
DS-HER: Decision support electronic health records
DSS: Decision support software
ECG: Electrocardiogram
EQ-5D 3 L: European Quality of Life 5 dimensions - 3 levels
GDP: Gross domestic product
HbA1c: Glycated haemoglobin
ICER: Incremental cost-effectiveness ratio
LDLc: Low-density lipoprotein cholesterol
LMIC: Low- and middle- income countries
QALYs: Quality adjusted life years
QI: Quality improvement
SBP: Systolic blood pressure
UK: United Kingdom
UKPDS: United Kingdom Prospective Diabetes Study
US$: United States dollar
